# Commensal Urinary Lactobacilli Inhibit Major Uropathogens *In Vitro* With Heterogeneity at Species and Strain Level

**DOI:** 10.3389/fcimb.2022.870603

**Published:** 2022-06-23

**Authors:** James A. Johnson, Lydia F. Delaney, Vaishali Ojha, Medha Rudraraju, Kaylie R. Hintze, Nazema Y. Siddiqui, Tatyana A. Sysoeva

**Affiliations:** ^1^ Department of Biology, University of Alabama in Huntsville, Huntsville, AL, United States; ^2^ Division of Urogynecology and Reconstructive Pelvic Surgery, Department of Obstetrics and Gynecology, Duke University, Durham, NC, United States

**Keywords:** urinary microbiome, commensal lactobacilli, urinary lactobacilli, urinary tract infection, uropathogens

## Abstract

The human urinary microbiome is thought to affect the development and progression of urinary tract infections (UTI), particularly recurrent UTIs in aging populations of women. To understand the possible interactions of urinary pathogens with commensal bacteria inhabiting the aging bladder, we conducted an initial functional assessment of a representative set of urinary lactobacilli that dominate this niche in postmenopausal women. We created a repository of urinary bladder bacteria isolated *via* Enhanced Quantitative Urinary Culture (EQUC) from healthy postmenopausal women, as well as those with a culture-proven recurrent UTI (rUTI) diagnosis. This repository contains lactobacilli strains from eight different species. As many other lactobacilli are known to inhibit human pathogens, we hypothesized that some urinary lactobacilli will have similar abilities to inhibit the growth of typical uropathogens and thus, provide a link between the urinary microbiome and the predisposition to the rUTI. Therefore, we screened the urinary lactobacilli in our repository for their ability to inhibit model uropathogens *in vitro*. We observed that many urinary isolates strongly inhibit model strains of gram-negative *Escherichia coli* and *Klebsiella pneumoniae* but demonstrate less inhibition of gram-positive *Enterococcus faecalis*. The observed inhibition affected model strains of uropathogens as well as clinical and multidrug-resistant isolates of those species. Our preliminary analysis of inhibition modes suggests a combination of pH-dependent and cell-dependent inhibition. Overall, inhibition strongly varies among species and strains of urinary lactobacilli. While the strength of the inhibition is not predictive of health outcomes in this limited repository, there is a high level of species and strain diversity that warrants future detailed investigations.

## Introduction

Urinary tract infections (UTIs) are among the most abundant bacterial infections worldwide, with over 6 million annual diagnoses in the U.S. alone (Flores-Mireles et al., 2015; Klein and Hultgren, 2020). The majority of UTIs are caused by enteric bacteria that may originate from the gastrointestinal tract, such as *Escherichia coli, Klebsiella pneumoniae*, and *Enterococcus faecalis* (Thomas-White et al., 2018). The rise of antibiotic-resistant uropathogens can make these infections difficult to treat and presents an urgent medical problem to which there is currently no sustainable solution. However, some individuals seem have a degree of protection against invasion by uropathogenic bacteria (Fouts et al., 2012; Brubaker and Wolfe, 2017; Klein and Hultgren, 2020), as predisposition to UTIs varies greatly between individuals. For instance, the prevalence of recurrent UTI (rUTI) is greatly increased in postmenopausal women (Flores-Mireles et al., 2015; Jung and Brubaker, 2019; Zeng et al., 2022).

Defined in the last decade, the urinary microbiome presents a diverse community of microorganisms, mainly bacteria, that are present at relatively low numbers in the urinary bladder (Wolfe et al., 2012; Ackerman and Underhill, 2017; Wolfe and Brubaker, 2019). Despite the low density of urinary microbes, compositional analyses using culture-independent methods such as 16S rRNA gene sequencing of different human cohorts reveal correlations between this microbiome and multiple urinary health conditions, including urinary urgency, incontinence, cancers, kidney stones, and recurrent UTIs (Fouts et al., 2012; Pearce et al., 2015; Whiteside et al., 2015; Thomas-White et al., 2017; Popović et al., 2018; Govender et al., 2019). Follow-up studies using a more advanced culturing technique—enhanced quantitative urinary culture (EQUC) (Hilt et al., 2014) —confirmed the presence of live bacteria that are typically not identified on a standard urine culture. Similar to the vaginal microbiome, the urinary microbiome often contains lactic acid-forming bacteria including lactobacilli (Thomas-White et al., 2018; Price et al., 2020a). The predominance of lactobacilli in the urinary microbiome or presence of particular lactobacilli species has been associated with protection against UTIs. In particular, some species, such as *Lactobacillus crispatus* and *Lactobacillis iners*, appear to correlate with urinary health (Pearce et al., 2014; Gottschick et al., 2017; Thomas-White et al., 2018; Neugent et al., 2020) but associations with recurrent UTI (rUTI) are less defined. Though a recent study has reported methods for distinguishing lactobacilli at a higher resolution using 16S rRNA gene sequencing, (Hoffman et al., 2021) the majority of urinary microbiome studies, to date, were conducted using an amplicon-based 16S rRNA gene sequencing method that is unable to distinguish lactobacilli at the species or strain levels.

Lactobacilli have been widely used for many years and are generally considered to be safe. Previously characterized lactobacilli, ranging from those used in food preparation to those isolated from the vaginal microbiome, have been investigated for their ability to inhibit the growth of other bacteria. Lactobacilli have been known to inhibit the growth of their competitors by producing organic acids, hydrogen peroxide, surfactants, and special toxins called bacteriocins (Jack et al., 1995; Zacharof and Lovitt, 2012; Mokoena, 2017). Lactobacilli also potentially contribute to probiotic functions. Indeed, many lactobacilli have been studied as potential non-antibiotic treatments to prevent recurrent UTI. However, these studies did not investigate lactobacilli species or strains isolated from the urinary tract (Stapleton et al., 2011; Grin et al., 2013; Gupta et al., 2017; Prabhurajeshwar and Chandrakanth, 2017; Smith et al., 2018; Neugent et al., 2020).

Urinary microbiome properties can provide explanations of observed differences in UTI susceptibility (Wolfe et al., 2012; Brubaker and Wolfe, 2017; Neugent et al., 2020), *via* interaction with the host, *via* the immune response, or *via* intermicrobial interactions. However, only minimal functional data are available for the commensal representatives of the human urinary microbiome. For example, recent examination of urinary isolates of *L. crispatus* found that they use phenyl-lactic acid to inhibit growth of uropathogens *E. coli* and *E. faecalis* (Abdul-Rahim et al., 2021). This shows similarity of the *L. crispatus* urinary strains with relatives from the vagina as well as diverse food sources (Jacobsen et al., 1999; Valerio et al., 2004; Hütt et al., 2016; Łaniewski and Herbst-Kralovetz, 2021). Additionally, mixed urinary bacterial communities from asymptomatic postmenopausal women and those with acute UTI were shown to interact differently, supporting the idea of complex intermicrobial interactions within the urinary microbiome and potential differential effects on UTI progression (Zandbergen et al., 2021).

We hypothesized that certain urinary lactobacilli will show a strong inhibition to uropathogen growth and thus provide protection against UTI in healthy individuals, while lactobacilli isolated from rUTI patients will not show a strong inhibition. To start addressing this hypothesis, we first collected a representative collection of culturable urinary bacteria from postmenopausal women with and without a history of recurrent UTI. Notably, all isolates were derived from urine in the absence of acute UTI. We then tested the ability of these urinary lactobacilli isolates as representatives of the commensal urinary microbiome to compete with the uropathogens.

## Materials and Methods

### Bacterial Growth and Preservation

Vaughan and coworkers (Vaughan et al., 2021) used published EQUC procedures (Hilt et al., 2014) to isolate urinary bacteria from catheterized urine specimens. Streak-isolated bacteria were typed by MALDI-TOF and then transferred to a set of liquid nutrient media: tryptic soy broth (TSB), Man Rogosa and Sharpe broth (MRS), and brain heard infusion broth (BHI). Inoculated media was statically incubated at 35°C for 24-72 hours with ambient or anaerobic atmospheric conditions. The isolates that did not produce enough biomass were inoculated in TSB supplemented with 5% sheep blood, cysteine (0.5 g/L), or other additives (hemin 5 mg/L, vitamin K 10 mg/L), or NYCIII medium (**Table S1**) and preserved as above if visible biomass was obtained. The qualitative densities of the resulting cultures were recorded and cell cultures were saved in 14% (w/v) glycerol at -80°C in duplicate freezer stocks. Later experiments with preserved strains showed that growth at 37°C worked similarly to 35°C and was used from then on.

### Complete Sanger Sequencing of the 16S rRNA Gene

To confirm species of urinary lactobacilli, we isolated genomic DNA of each used strain from overnight MRS culture using the Qiagen PowerSoil kit. Then we amplified most of the 16S rRNA gene using classic primers 27F (5’-AGAGTTTGATCCTGGCTCAG-3’) and 1492R (5’-GGTTACCTTGTTACGACTT-3’) and Q5 polymerase mastermix (NewEngland Biolabs). The resulted PCR product was purified and submitted for Sanger sequencing (Genewiz).

### Well-Diffusion Inhibition Assays

Inhibition of a number of substances was measured *via* a well-diffusion assay using solid MRS agar plates. Lactobacilli strains were grown over two nights in 10 mL of MRS broth and uropathogenic strains were grown overnight in 4 mL of LB broth, both stationary at 37°C. Surface of the MRS agar plates was then inoculated with 100 μL of the uropathogenic culture. Wells were punched in the surface (Ø6 mm) and removed with sterile tweezers. 50 μL of the bacterial culture or filtrates to be tested were added to the wells. The plates were then grown overnight at 37°C, unless mentioned otherwise. To measure the size of the zones of inhibition, all plates were first imaged, and the zones were quantified by the graphical program GIMP (www.gimp.org) and analyzed in MS Excel.

### Liquid Inhibition Assays

As in the solid agar inhibition assays, lactobacilli strains were grown for two nights in 10 mL of MRS broth and uropathogenic strains were grown overnight in 4 mL of LB broth. The optical density as measured at 600 nm (OD_600_) of the strains was then taken, and 20 μL of the uropathogen culture was placed into each of the tubes needed for the experiment. The OD_600_ was then used to calculate the amount of lactobacilli culture needed to make specific ratios of uropathogen: lactobacilli and that amount of lactobacilli culture was added to their respective tubes. These tubes were then spun in a centrifuge at 3230 rcf for 10 minutes to form a cell pellet and the supernatant was poured off. The pellets were then resuspended in 10 mL of MRS broth and incubated at 37°C. Measurements of uropathogen density were taken *via* serial dilutions and colony counting and samples were taken at four time points after beginning incubation: 0 hours, 1 hour, 6 hours, and 24 hours. At each of these time points, 100 μL was taken from each tube and used to make serial dilutions ranging from undiluted to a dilution of 10^-7^. From each of these, 2 μL was taken and plated in rows on LB agar plates which were then incubated for several hours at 37°C. The number of colonies in least diluted but still countable samples was then used to calculate the CFU/mL of *E. coli* in each tube.

### Spent Media Manipulations

In several experiments, cells were removed from the culture to isolate the effects of the cells from the effects of cell secretions in the media. This filtrate was made by first spinning a few milliliters of the culture in a centrifuge at 3230 rcf for 10 minutes. The supernatant was then poured into a syringe and pushed through a 0.22 μm PES filter into a sterile tube. This filtrate was then ready for use or further modification as needed for the experiment.

### Statistical Analysis

All experiments were done at least in triplicate, with well-diffusion assays being run with technical triplicates on three separate days to represent biological replicates. All p-values were determined by a two-tailed Student’s T-test to determine statistical differences between different groups. We used a significance level of 0.05 and p-values of 0.01 or less are marked as well.

## Results

### Establishing a Repository of Urinary Bacteria

A new urinary bacteria repository was created following the recent study by Vaughan and colleagues (Vaughan et al., 2021). In that study, asymptomatic postmenopausal women provided catheterized urine that was then processed with 16S rRNA amplicon-based gene sequencing and EQUC. Three cohorts of women using vaginal estrogen therapy were tested: those without rUTI [noUTI]; those with rUTI [rUTI]; and those with rUTI and also taking daily prophylactic antibiotics as part of their treatment regimen [rUTI+ab]. Full details on these cohorts including exclusion criteria and demographic information are published elsewhere (see **Tables 1**, **2** of the prior report) (Vaughan et al., 2021). Bacteria isolated using EQUC were typed using mass spectrometry (MALDI-TOF) showing that the collection consists of 64 microbial strains from 24 species of bacteria and two species of fungi (**Tables 1** and **S1**). A subset of lactobacilli was confirmed using Sanger sequencing (**Table S2**). While in 2020 the *Lactobacillus* genus was reclassified into 25 separate genera (Zheng et al., 2020). We have representatives of three of them and will refer to them collectively as lactobacilli throughout the manuscript for simplicity.

**Table 1:** Urinary Isolates repository.

Species	Number Identified
***Lactobacilli***	*27*
*Lactobacillus acidophilus/gasseri*	8
*Lactobacillus casei/rhamnosus/paracasei*	5
*Lactobacillus crispatus*	5
*Lactobacillus delbrueckii*	4
*Lactobacillus iners*	1
*Lactobacillus jensenii*	2
*Lactobacillus spp*	2
**Strains from well-established uropathogen species**	14
*Escherichia coli*	5
*Klebsiella pneumoniae*	3
*Klebsiella oxytoca*	1
*Proteus mirabilis*	1
*Enterococcus faecalis*	4
**Strains from commensal and opportunistic pathogen species**	24
*Corynobacerium coyleae*	1
*Staphylococcus epidermidis*	4
*Staphylococcus hominis*	1
*Staphylococcus ludgenensis*	1
*Streptococcus anginosus*	6
*Streptococcus bovis group*	1
*Streptococcus sanguinis alpha*	1
*Streptococcus sp*	1
*Aerococcus urinae*	3
*Leuconostoc sp*	1
*Raoultella ornithinolytica*	1
Diphtheroids	3
**Others (fungi)**	3
*Candida albicans*	2
*Candida glabrata*	1

Identities of isolated microbes in the repository, as determined by MALDI-TOF typing.

**Table 2:** Strains of uropathogen species used in this study.

Species	Strain	Notes/Phenotypes	References/Source
*E. coli*	MG1655	Non-pathogenic strain	Lab stock
	CFT073	UTI model (urosepsis isolate)	(Mobley et al., 1990); Mehreen Arshad, Northwestern University
	DS17	UTI model (pyelonephritis isolate)	(Tullus et al., 1984); Mehreen Arshad, Northwestern University
	UTI89	UTI model (cystitis isolate)	(Mulvey et al., 2001); Mehreen Arshad, Northwestern University
	ESBL41	MDR clinical isolate	(Kanamori et al., 2017; Bethke et al., 2020); Deverick Anderson, Duke University
	ESBL146	MDR clinical isolate	(Kanamori et al., 2017; Bethke et al., 2020); Deverick Anderson, Duke University
	ESBL168	MDR clinical isolate	(Kanamori et al., 2017; Bethke et al., 2020); Deverick Anderson, Duke University
	ESBL193	MDR clinical isolate	(Kanamori et al., 2017; Bethke et al., 2020); Deverick Anderson, Duke University
*K. pneumoniae*	TOP52	UTI model (cystitis isolate)	(Rosen et al., 2007; Johnson et al., 2014); Jyl Matson, University of Toledo
*E. faecalis*	OG1RF	UTI model	Juliett Willett and Gary Dunny, University of Minnesota
	JH2-2	UTI model	Juliett Willett and Gary Dunny, University of Minnesota
	V583	MDR strain	Juliett Willett and Gary Dunny, University of Minnesota
	MMH594	MDR strain	Juliett Willett and Gary Dunny, University of Minnesota

Consistent with many urinary microbiome studies, almost half of this collection contains lactobacilli from eight species: *Lactobacillus gasseri, Lactobacillus delbrueckii, Lacticaseibacillus rhamnosus, Ligilactobacillus animalis, Lactobacillus jensenii, Lactobacillus jonsonii, L. iners*, and *L. crispatus*. Due to the nature of the EQUC procedure, all these strains are aerotolerant and our tests showed that the majority of them grow sufficiently well in the presence of oxygen but without active mixing. The exceptions were *L. iners*, some *L. crispatus*, and a few other bacterial isolates which required specialized media or were particularly sensitive to oxygen changes (**Table S1**). Predictably, the rest grew well in MRS broth designed for lactobacilli growth.

It is worth noting that the repository also contains 5 uropathogen species derived in the same fashion from the same research participants: *E. coli, K. pneumoniae*, *K. oxytoca*, *Proteus mirabilis*, and *E. faecalis* (**Tables 1** and **S1**). As all samples were derived in the absence of acute UTI and at least 4 weeks after treatment for UTI (Vaughan et al., 2021), it is unclear if these strains were present in the setting of different host immune tolerance mechanisms and if these strains had adapted to survive within the normal urinary microbiome. It is also possible, as recently reported, that similar to the vaginal tract, low level presence of pathogenic bacteria in the bladder is common (Thomas-White et al., 2018; Garretto et al., 2020; Price et al., 2020b).

### Two Initial Urinary Lactobacilli Isolates Inhibit Growth of Model Uropathogens

**Figure 1:**
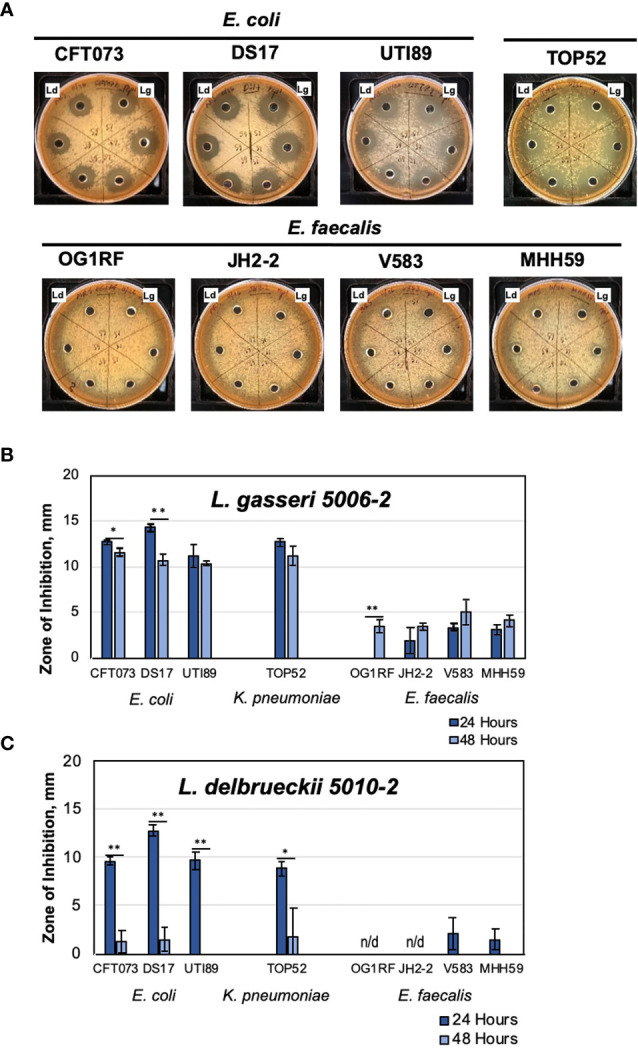
Urinary lactobacilli *L. gasseri* 5006-2 and *L. delbrueckii* 5010-2 inhibit a broad range of uropathogens. Representative images of well-diffusion inhibition assays using *E. coli, K. pneumoniae*, and *E. faecalis* **(A)**, and zone of inhibition sizes of one-day old (dark blue) or two-day old (light blue) cultures of *L. gasseri* 5006-2 [Lg**, (B)**] and *L. delbrueckii* 5010-2 [Ld, **(C)**] against 8 pathogenic strains (**Table 2**). Error bars indicate standard deviation of three biological replicates, *p < 0.05 and **p < 0.01.

At the beginning of the EQUC collection, we established the whole genome sequences of two initial isolates of urinary lactobacilli – *L. gasseri 5006-2* (GenBank Accession code JAGEKM000000000.1) and *L. delbrueckii 5010-2*. These two strains come from healthy, postmenopausal women and one with a history of rUTI, respectively. Preliminary genome analysis showed the presence of several bacteriocin genes. In light of these preliminary findings, we sought to assess whether major urinary pathogens can be inhibited by these urinary lactobacilli. We used model UTI strains of three major uropathogens: *E. coli, K. pneumoniae*, and *E. faecalis* (**Table 2**) and tested the two sequenced lactobacilli strains for inhibition of pathogens using well-diffusion assays on MRS agar plates (**Figure 1A**).

Lactobacilli strains were first competed against three different uropathogenic strains of *E. coli*, CFT073, DS17, and UTI89, in a well-diffusion assay (**Figure 1A**) (Tullus et al., 1984; Mobley et al., 1990; Mulvey et al., 2001). Both lactobacilli strains inhibited the growth of all *E. coli* strains, though *L. gasseri* generally showed more inhibition than *L. delbrueckii*. One notable difference between the two strains can be seen in their inhibition when grown for 48 hours, rather than only 24 (**Figures 1B, C**). An *L. gasseri* culture grown for 24 hours seems to have slightly better inhibition of *E. coli* strains than those grown for 48 hours. Cultures of *L. delbrueckii* grown for 24 hours inhibited *E. coli* growth at comparable, albeit slightly lower, levels than the other lactobacilli strain; however, this inhibition dropped massively when cultures were grown for 48 hours, and in some cases nearly to zero. The timing of inhibition appeared to be sensitive to properties of the initial inoculum of *L. delbrueckii* strain.

The inhibition was next tested against other common UTI pathogens – *K. pnemoniae* and *E. faecalis*. For these tests, one strain of *K. pneumoniae* (TOP52) and four strains of *E. faecalis* (OG1RF, JH2-2, V583, and MHH59) were used (**Figures 1B, C**). Significantly, we observed some level of inhibition against all strains tested, even the Gram-positive *E. faecalis* strains (**Figure 1A**). The *K. pneumoniae* strain was sucessfully inhibited by both lactobacilli strains and the amount of inhibition observed was similar to assays performed using *E. coli* UTI model strains. *L. gasseri* cultures grown for both 24 and 48 hours were able to inhibit TOP52. Cultures of *L. delbrueckii* were able to inhibit TOP52 when grown for 24 hours but not when grown for 48 hours. The *E. faecalis* strains were not inhibited nearly as much as the gram-negative strains; however, they were all inhibited to some degree. *L. gasseri* cultures, in particular, were able to inhibit all *E. faecalis* strains to a limited degree after growth for 48 hours and all but OG1RF was inhibited after 24 hours of growth. The *L. delbrueckii* strain, however, was only able to inhibit two of the *E. faecalis* strains at a very low level when grown for 24 hours.

Such inhibition tests were then repeated with antibiotic resistant strains of *E. coli* (ESBL41, ESBL 146, ESBL146, and ESBL193 (Kanamori et al., 2017; Bethke et al., 2020). We determined that lactobacilli inhibition extended to antibiotic resistant *E. coli* as well (**Figure S2**). Notably, tested model *E. faecalis* V583 and MHH95 are also multidrug resistant and can be inhibited by *L. gasseri 5006-2* (**Figure 1**).

Overall, these tests show that the two selected lactobacillus strains were able to consistently inhibit several model uropathogens, potentially indicating a general inhibitory mechanism. These preliminary observations allowed us to set up feasible methods to screen the majority of urinary lactobacilli from our repository under the same growth conditions.

### Majority of Tested Strains From Seven Urinary Lactobacilli Species Inhibit the Growth of Model Uropathogens

**Figure 2:**
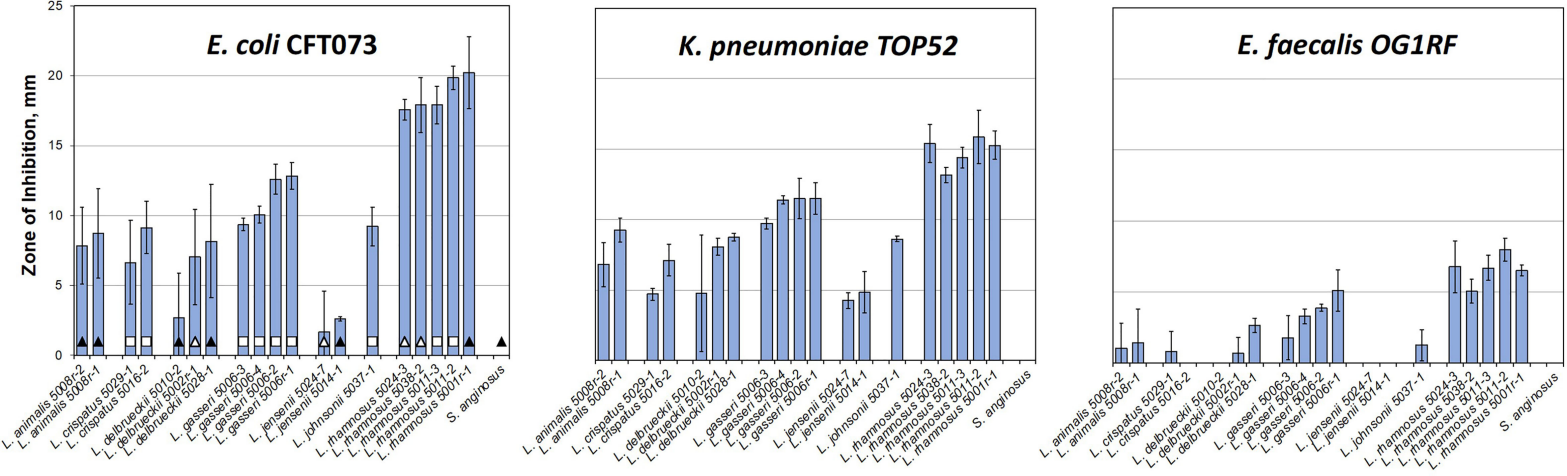
Majority of urinary lactobacilli exhibit varying degrees of inhibition of major uropathogens. Twenty strains of urinary commensal lactic acid bacteria (lactobacilli and one strain of *Streptococcus anginosus* as a control) were tested against model uropathogenic strains: *E. coli* CFT073, *K. pneumoniae* TOP52, and *E. faecalis* OG1RF. Bars indicate the standard deviation of three replicates. The leftmost plot carries identifications of the patient cohorts for each strain (**Table S3**): empty square – no UTI; black triangle – rUTI; white triangle – rUTI with prophylactic antibiotics. The data are presented by species alphabetically and sorted by the inhibition against CFT073 within species set.

Our initial tests showed that urinary *L. gasseri 5006-2* and *L. delbrueckii 5010-2* strains can inhibit uropathogens *in vitro*. Moreover, the strength of the inhibition was coincidental with the rUTI status of the patients from whom the strains originated (**Table S3**). We therefore set to test other strains from the same species and five other lactobacilli species in our repository that can be grown under the same conditions. This selection of the lactobacilli species, that were grown under similar conditions, left out several strains of *L. crispatus* and one *L. iners* strain. Overall, we screened 19 lactobacilli strains grown for 24 and 48 hours in static MRS broth. The *Streptococcus anginosus* 5008-3 strain was used as a control for unrelated lactic acid bacteria that are also frequently found in the lower urinary tract (**Table 1**, **S1**). This was done by using a well-diffusion assay against 3 model uropathogens: *E. coli* CFT073, *K. pneumoniae* TOP52, and *E. faecalis* OG1RF (**Figure 2**).

Our inhibition data show that the amount of inhibition varied more among different species of lactobacilli than it did among strains within the species. Unfortunately, with only one to five representatives from each species, it is impossible to generalize, apart from recording that the differences between strains and species in our subset are notable. For instance, the *L. rhamnosus* strains all have similarly sized, large zones of inhibition, especially towards the two gram-negative uropathogens. The *L. jensenii* strains did not show a strong inhibition under conditions tested with no detectable inhibition zone on the *E. faecalis* plate. The *L. crispatus*, *L. gasseri*, *L. delbrueckii*, *L. jonsonii*, and *L. animalis* strains all showed intermediate inhibition levels. It is noteworthy that testing 24 and 48 hr. old lactobacilli cultures (**Figure S3**) showed significant differences in inhibition strengths, with *L. crispatus*, *L. delbruecki* and *L. jensenii* being particularly sensitive to lactobacilli culture growth phase.

As the isolates in our repository came from three different cohorts of patients (**Table S2**), we compared the strength of lactobacilli inhibition and cohort status with the hypothesis that there would be greater inhibition in lactobacilli derived from healthy women without rUTI. The presented screen shows that there is no correlation between the patient cohort and the ability of EQUC isolated lactobacilli to inhibit uropathogens *in vitro* (**Figure 2** and **Figure S4**). No statistically significant differences among cohorts are observed for such interspecies or averaged inhibition against either of the three uropathogens (**Figure S4**).

In summary, results show that inhibition is widespread across urinary lactobacilli species, but show heterogeneity among different uropathogens and urinary lactobacilli.

### Co-Existence of Antagonistic Species in the Same Bladder

**Figure 3:**
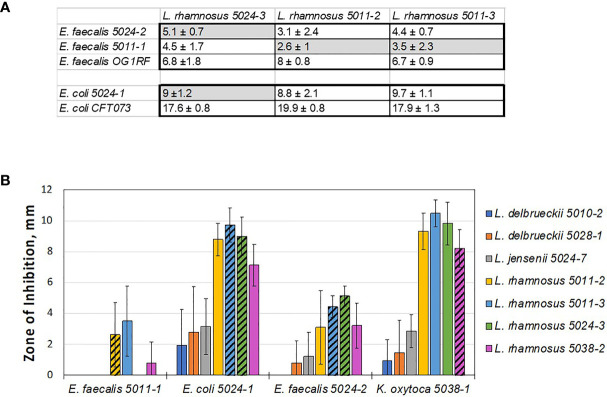
Lactobacilli cohabiting the same bladder with a pathogen still can inhibit that pathogen *in vitro*. **(A)** Table showing average zones of inhibition (mm) by cohabiting *E. coli* and *E. faecalis* strains. The comparison indicates that cohabiting pathogen strains (shaded in grey) are inhibited by the respective cohabiting commensal urinary lactobacilli but to lesser degree than other pathogen isolates and the model uropathogen strains (no grey shading). **(B)** Overall screen of patient isolates shows that ‘better inhibiting’ lactobacilli strains against one pathogen isolate also show ‘better inhibiting’ against another pathogen isolate. In addition, *L. rhamnosus* strains appear to be better inhibitors, similar to the model screen (**Figure 2**).

In some of the collected urines, the EQUC method isolated multiple species co-existing in the same sample, including both lactobacilli and uropathogens (**Table S1** and **S3**). As noted, none of the women who provided urine had acute symptomatic UTI and all underwent screening with dipstick urinalysis confirming the absence of pyuria prior to providing research specimens. Nevertheless, the *in vivo* coexistence of uropathogens with lactobacilli species seem to counter the simple hypothesis that presence of uropathogen-inhibiting lactobacilli should eliminate survival of uropathogens in the same niche. For this reason, we sought to test the inhibition of these pathobiont isolates with their cohabitating lactobacilli species to see if lactobacilli that are cohabitating with uropathogens are less antagonistic. To do this, we set up a broad screen to test each of the lactobacilli species isolated from these women (**Table S3**) for inhibition of each of the pathobiont species isolated from that woman’s same sample (**Figure S5**). Unfortunately, the *K. pneumoniae* isolates failed to form robust lawns on the test MRS agar plates and therefore, were not included in this cohabitant’s screen. Otherwise, all combinations of *E. coli*, *E. faecalis*, and *K. oxytoca* (**Figure S5**) with lactobacilli were measured. To our surprise, most of the measured pairs showed some levels of inhibition, including strain combinations isolated from the same individual at the same time (**Figure 3** and **Table S3**).

We then compared the ability of cohabitating lactobacilli strains to inhibit model uropathogen *E. coli* or *E. faecalis*, strains of the same uropathogen species that coexisted with the given lactobacilli strain, or were isolated from other individuals. From this small subset it appears that the cohabiting *lactobacillus* strain did not inhibit uropathogen isolates as strongly as the inhibition noted previously with model uropathogens (OG1RF and CFT073, **Figure 3A**). With no further genetic or pathogenicity information available for these new isolates, it is not clear if this is due to properties of the lactobacilli or uropathogen strain.

While our data on cross-competing novel clinical isolates against each other, cohabiting or not, it does not support the hypothesis that *in vitro* inhibition can predict *in vivo* behavior – it again shows how the inhibitory interaction is specific and sensitive to the identity of the lactobacilli used at a strain-level. Nevertheless, we do observe that some species tend to be better inhibitors on a relative scale, when compared to others. Specifically, *L. delbrueckii 5010-2, L. delbrueckii 5028-1*, and *L. jensenii 5024-7* did not inhibit well in a screen with model uropathogens and they consistently exhibit less inhibition with uropathogen isolates derived clinically from patients (compare **Figures 1**-**3**).

The results of testing urinary lactobacilli and pathobionts found in the same urine sample indicate that antagonistic species might coexist *in vivo*.

### Interference of Urinary Lactobacilli With Uropathogens in Liquid Media

While informative, simple inhibition on agar plates is not be reflective of the physiological conditions of the lower urinary tract. Therefore, we tested the interactions of urinary lactobacilli and UPEC in liquid MRS media, which might provide a better, though still not perfect, representation of bacterial suspension in urine within the lower urinary tract. We therefore co-cultured *E. coli* CFT073 with increasing amounts of *L. gasseri 5006-2* or *L. delbrueckii 5010-2* in liquid growth media.

**Figure 4:**
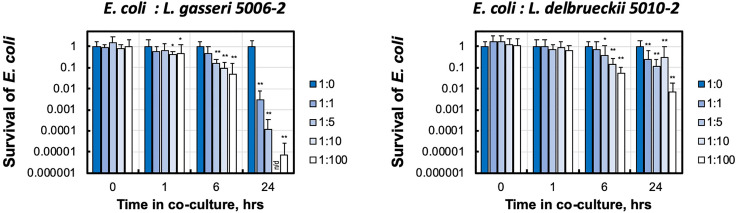
Urinary lactobacillus strains inhibit *E. coli* growth when co-cultured in liquid medium. Uropathogenic *E. coli* CFT073 was grown in presence of increasing amounts of initial lactobacilli (*L. gasseri* 5006-2 and *L. delbrueckii* 5010-2) with ratios 1:0, 1:1, 1:5, 1:10, and 1:100 estimated by the OD600 measurements. *E. coli* survival was measured by LB plating of serial dilutions for CFU counting and compared with growth of *E. coli* only culture (1:0). Survival is calculated as culture density normalized to *E. coli* only culture. Error bars show only positive arm of standard deviation for three biological replicates. *p < 0.05 and **p < 0.01 and indicate a significant decrease from *E. coli* grown in the presence of lactobacilli. Over the course of this experiment *E. coli* for lactobacilli-free condition (1:0) grows from ~ 2.5-3.4×10^6^ to ~ 8.4 - 9.7×10^8^ CFU/mL densities. n/d – the limit of detection of our CFU counting was at about 500 CFU/mL, corresponding to ~ 0.5×10^-6^ survival.

The presence of either of these lactobacilli strains dramatically decreased the growth of the *E. coli* CFT073 in media (**Figure 4**). This can be seen in the significantly reduced amount of *E. coli* after only 6 hours of growth together with either strain of lactobacilli. After 24 hours, both *L. delbrueckii 5010-2* and *L. gasseri 5006-2* were able to reduce CFT073 densities by two to five orders of magnitude. Cultures containing the *L. gasseri 5006-2* strain, similar to results in the well-diffusion assays, were able to inhibit to a greater degree than *L. delbrueckii 5010-2* cultures.

These experiments showed that the dynamics and strength of inhibition in liquid is different between the lactobacilli strains.

### Mechanism of Uropathogen Inhibition by Select Lactobacillus Species

**Figure 5:**
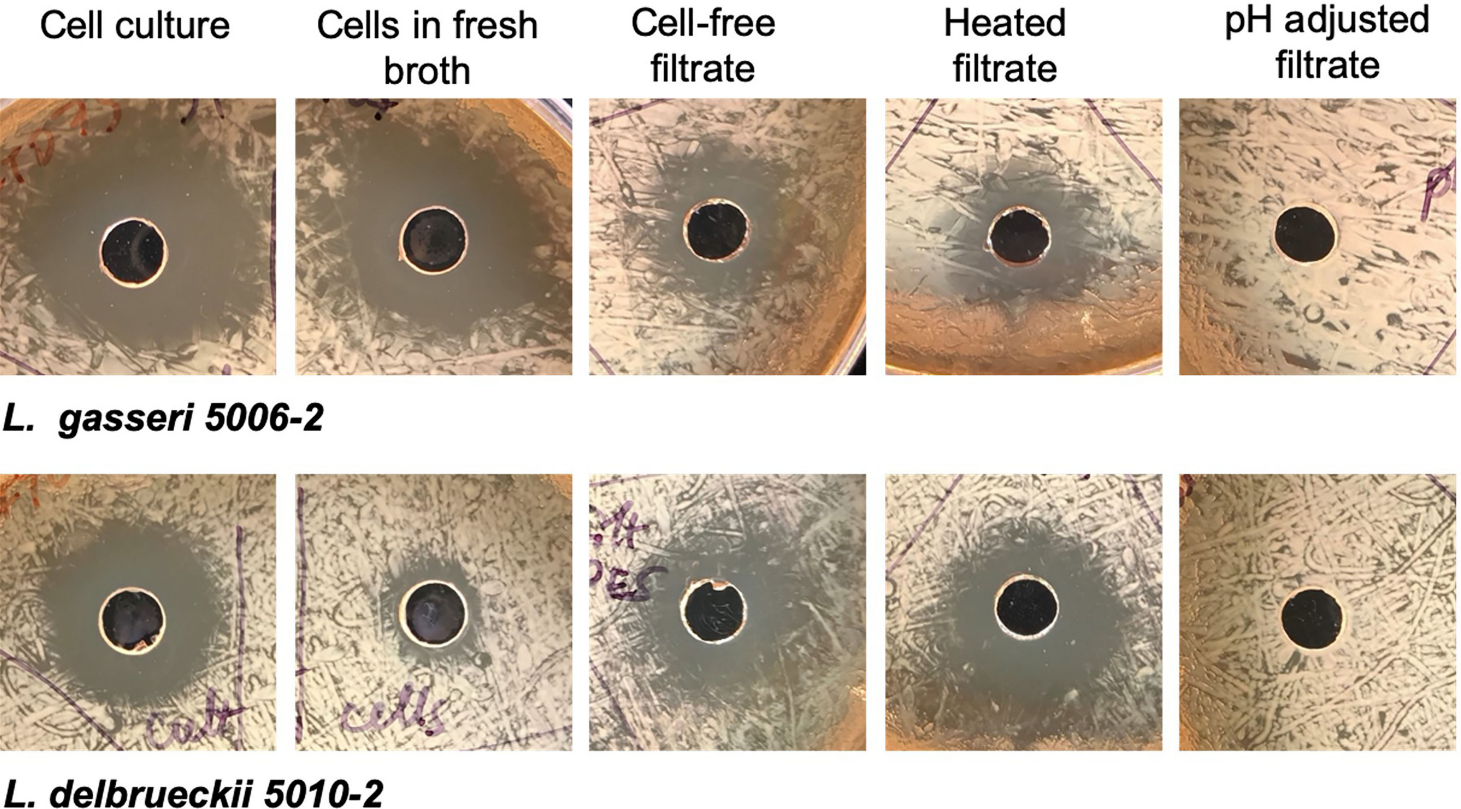
Lactobacilli inhibition differs depending on culture treatment. Representative images from well-diffusion assays using whole cell culture, cells resuspended in fresh broth, cell-free filtrate, heated filtrate, and filtrate adjusted to a neutral pH. Images taken for 24 hrs. growth at 37°C.

To determine how the inhibition took place, lactobacilli *L. gasseri 5006-2* and *L. delbrueckii 5010-2* cultures were split into two parts: a cell-free filtrate and a suspension of cells in fresh broth. The filtrate of both lactobacilli cultures could inhibit the growth of the *E. coli* CFT073 strain starting after about 12 hours of incubation together on a plate and lasting until about 24 hours of incubation together (**Figure 5**). However, this inhibition seems to be somewhat transient as when plates were incubated for longer than 24 hours, the *E. coli* would begin overgrowing the zone of inhibition, causing the clearance zone to shrink until it vanished in ~ 4 days (**Figure S6**). Zones from the resuspended cells, in contrast, differed markedly between the two lactobacilli cultures. Resuspended *L. delbrueckii 5010-2* cells exhibited some inhibition, but usually not as strong as that of cell-free filtrate or *L. gasseri 5006-2* strain (**Figure 5**). Resuspended *L. gasseri 5006-2* cells showed inhibition comparable to that of the respective whole culture. In addition, the zones of inhibition from resuspended cells of both strains did not change if the plates were incubated longer than the standard 24 hours while filtrate-induced inhibition zones were eventually overgrown by *E. coli* lawn (**Figure S6**). This seems to indicate an additional mechanism of inhibition present in the *L. gasseri* cultures; one that is dependent on the presence of cells and which does not seem to be resulting from acidification. This seems to show that both lactobacilli species inhibit uropathogens by decreasing the pH of their environment and the *L. gasseri* strain may have a strong additional cell-based mechanism of action that is independent of a low pH.

The cell-free filtrates from both *L. delbrueckii* and *L. gasseri* cultures were then heated or adjusted to a neutral pH before use in a well-diffusion assay. The filtrate showed the same amount of inhibition when heated as it did when unmodified, indicating that the mechanism of inhibition is resistant to heat. The filtrate from both cultures that was adjusted to a neutral pH, conversely, showed no inhibition. In addition to neutralizing the filtrate, another experiment increased the buffering capacity of the medium used in the experiment by adding PBS to the MRS agar formulation. These plates showed significantly reduced inhibition by filtrates from both lactobacilli cultures. Finally, pH readings of the filtrate before use in well-diffusion assays seem to indicate that a low pH (~3.9-4.0) is needed for inhibition to occur. Taken together, this seems to indicate that the mechanism of inhibition in the cell-free filtrate is highly dependent on pH and is likely a result of the acidification of their environment by lactobacilli species.

One possible explanation for cell-dependent inhibition is the presence of biosurfactants. Several reports showed that phosphate-buffers can elute such surfactants into solution (Rodrigues et al., 2006; Gudiña et al., 2011; Morais et al., 2017). To test for this hypothesis, cells were incubated in PBS for varying lengths of time (1-24 hr) to isolate any biosurfactant molecules, then the PBS solution was filter-sterilized, and used in well-diffusion assays. However, the PBS incubated with either of the strains’ cultures was not able to inhibit CFT073, indicating a lack of inhibitory biosurfactants that can be eluted this way (*data not shown*).

## Discussion

In this study, we conducted initial functional characterization of urinary lactobacilli from seven species under uniform conditions. Three of those species (eight strains) are from the abundant and frequently identified species in the female urinary tract: *L. crispatus, L. gasseri* and *L. jensenii* (Ma et al., 2012; Komesu et al., 2020; Neugent et al., 2022). While lactobacilli inhabiting other ecological niches have been previously studied, urinary lactobacilli have not previously been characterized, despite their potential for direct interactions with uropathogens and potential clinical implications regarding urinary tract infections.

We found that most urinary lactobacilli species strongly inhibit the growth of gram-negative *E. coli* and *K. pneumoniae*, including model strains and drug-resistant clinical isolates. Many of these same strains, however, either showed modest levels of inhibition or did not inhibit the growth of the gram-positive *E. faecalis*. *L. gasseri 5006-2* and *L. delbrueckii 5010-2* inhibited the growth of uropathogenic *E. coli* both on solid agar and in liquid media (**Figures 1**–**4**). Such inhibition is relevant to bacterial interaction at the urothelial surface and in bulk urine, respectively.

Our study shows that there is high variability of inhibition strength among lactobacilli species and sometimes strains of the same species. The data for *L. gasseri* and *L. rhamnosus* species identifies them as strong inhibitors of uropathogens *in vitro*. *L. delbrueckii* is a less efficient inhibitor. The *L. jensenii* and *L. crispatus* strains tested show only modest inhibition in our assays.

Interestingly, *L. crispatus* has been previously associated with a healthy host urinary tract in some urinary microbiome studies (Pearce et al., 2014; Neugent et al., 2022) but no difference in abundance of this species was found in others (Komesu et al., 2020; Vaughan et al., 2021). Urinary *L. crispatus* isolates recently were found to inhibit *E. coli* CFT073 and *E. faecalis* in TSB medium *via* accumulation of phenyl-lactic acid (Abdul-Rahim et al., 2021). On the contrary, the urinary strain of *L. gasseri UMB4205* tested by Abdul-Rahim and colleagues was not inhibiting *E. coli* growth on TSB agar (Abdul-Rahim et al., 2021). These differences may confirm the overall heterogeneity in the urinary lactobacilli phenotypes we observed or may reflect on the sensitivity to the culture testing conditions among the studies. It is also plausible that different phenotypes are characteristic if isolates are derived from the urinary bladder in different age groups or health conditions and thus, findings from individual species may not be comparable ‘across the board’.

Similar heterogeneity in phenotypes was previously found for vaginal lactobacilli. For example, vaginal *L. crispatus* isolates were shown to be stronger inhibitors of *E. coli* and candida than *L. jensenii* and *L. gasseri* in a study by (Hütt et al., 2016). Other screening of vaginal *L. gasseri* and *L. crispatus* showed strong differences in some antimicrobial activities, but not others (Atassi et al., 2019).

The inhibition heterogeneity and additional testing of the two isolates (*L. gasseri* and *L. delbrueckii*) suggest that the underlying inhibition mechanisms are complex. At least for *L. gasseri* 5006-2, inhibition has both pH-dependent and cell-dependent components (**Figure 5**). Acidification of the environment by abundant lactobacilli is thought to be the main factor resisting pathogens in the vagina. However, in contrast to the vagina, the bladder microbiome cannot have overall high lactobacilli density. In addition, bulk urine pH is changing with metabolism and diet making it unlikely to maintain an overall low urine pH. Nevertheless, it is possible that in some niches where lactobacilli adhere, they can cause local acidification and thus, adjacent protection against uropathogens.

While it is tempting to look for simple differences in binary inhibitory interactions of urinary lactobacilli with uropathogens to explain propensity towards rUTI, our data did not show correlations between strength of lactobacilli inhibition and patient cohorts (**Figure 2** and **S4**). This is consistent with the lack of strong correlations in postmenopausal urinary microbiome composition with rUTI status (Vaughan et al., 2021). Moreover, the observed inhibition of lactobacilli against the growth of pathogens found in the same urine samples rules out the hypothesis that inhibition of a uropathogen by present lactobacilli will automatically eliminate the uropathogen. These observations are not entirely unexpected, as lactobacilli and uropathogen are only a part of a complex urinary microbiome in which presence of other bacteria might assist or negate the inhibitory lactobacilli effects.

Even binary interaction between lactobacilli and *E. coli* uropathogen might include aspects other that inhibition by lactobacilli. For example, there is a known example of an interaction in which presence of uropathogens might enhance the survival of auxotrophic lactobacilli, similar to those found in the bladder, in minimal medium M9 (Mizuno et al., 2017). *E. coli* was also found to co-aggregate with several lactobacilli (Mizuno et al., 2014). In addition, adhesion of lactobacilli to gut and vaginal epithelium was shown to inhibit pathogen adhesion (Osset et al., 2001; Otero and Elena Nader-Macías, 2007; Wang et al., 2018; He et al., 2020) and such effects can be applicable to the urinary tract.

While we tested several isolates of lactobacilli, it possible that any isolates from postmenopausal women are less protective *in vivo* against UTI, potentially explaining the documented susceptibility of this patient population to rUTI (Pearce et al., 2014; Komesu et al., 2020; Vaughan et al., 2021; Neugent et al., 2022; Zeng et al., 2022). Therefore, to advance our understanding of the urinary microbiome interactions with uropathogens, it will be essential to compare the behavior of urinary lactobacilli from healthy and rUTI cohorts of pre-menopausal women. Nevertheless, the presented characterization of urinary lactobacilli here, from postmenopausal women with and without rUTIs, is critical to our understanding of how lactobacilli function within the postmenopausal urinary tract. These findings also provide inferences for which lactobacilli probiotic candidates can stably colonize the niche.

In liquid inhibition, the results indicated that the ratio between the uropathogen and commensal is important for efficient inhibition over time (**Figure 4**). Thus, the future studies need to identity uropathogen(s) in a given rUTI patient to establish densities and distribution of the lactobacilli and uropathogen cells and to characterize the dynamics of this complex community. Without the detailed data, we cannot distinguish situations in which rUTI patients may continue to remain asymptomatic due to urinary lactobacilli, keeping uropathogen populations ‘in check’ or in which urinary lactobacilli simply cannot inhibit the growth of the uropathogens.

In conclusion, our study shows that urinary lactobacilli behave like lactobacilli isolated from other sources and exhibit the strong ability to inhibit the growth of different uropathogens. This inhibition is not limited to classic acidification of media and broadly differs among the species and strains inhabiting the bladder, uropathogen type, and conditions of the lactobacilli growth. These results highlight the necessity of detailed investigations of the composition of the urinary microbiome at the species and sub-species resolution level and careful functional characterization of urinary microbial representatives, separately and in communities. These studies will bring us closer to understanding UTI progression and recurrence and to developing UTI-targeting personalized pre- and probiotics.

## Data Availability Statement

The original contributions presented in the study are included in the article/**Supplementary Material**. Further inquiries can be directed to the corresponding author.

## Author Contributions

Conceptualization and method development: TS. Experimental design and performance: JJ, TS, MR, VO, LD, and KH. Repository creation: NS and TS. Data analysis: JJ and TS. Funding acquisition and project management: TS. Manuscript writing and editing: JJ, NS, and TS. All authors contributed to the article and approved the submitted version.

## Funding

This study was in part sponsored by K12 Duke KURe (DK100024 NIDDK) and UAH Startup funds to TS.

## Conflict of Interest

The authors declare that the research was conducted in the absence of any commercial or financial relationships that could be construed as a potential conflict of interest.

## Publisher’s Note

All claims expressed in this article are solely those of the authors and do not necessarily represent those of their affiliated organizations, or those of the publisher, the editors and the reviewers. Any product that may be evaluated in this article, or claim that may be made by its manufacturer, is not guaranteed or endorsed by the publisher.
