# Supplementary material for: Commensal Urinary Lactobacilli Inhibit Major Uropathogens In Vitro With Heterogeneity at Species and Strain Level
Source: Front Cell Infect Microbiol. 2022 Jun 23;12:870603. doi: 10.3389/fcimb.2022.870603 (PMC9260849; doi:10.3389/fcimb.2022.870603)
Supplement: Supplementary file 1 [file DataSheet_1.pdf]

Supplemental Materials

Manuscript "Commensal urinary lactobacilli inhibit major uropathogens *in vitro* with heterogeneity at species and strain level" by
James A. Johnson, Lydia F. Delaney, Vaishali Ojha, Medha Rudraraju, Kaylie R. Hintze, Nazema Y. Siddiqui, Tatyana A. Sysoeva

Table S1. EQUC Urinary Isolates Repository

ID	Isolate ID	MALDI typing	EQUC plate	Liquid medium	T, °C	Aerobic	TSB	BHI	MRS
5001	5001-1	*Lactobacillus sp (casei, rhamnosus,paracasei)*	Sheep Blood	BHI+yeast extract,cysteine	35	Y	-	with additives	-
5002	5002-1	*Lactobacillus delbrueckii*	Sheep Blood	MRS	37	Y	+	-	+++
5006	5006-1	*Streptococcus anginosus*	Sheep Blood	TSA+yeast extract,cysteine	35	Y	with additives	-	-
	5006-2	*Lactobacillus sp (gasseri,acidophilus)*	Sheep Blood	MRS	35	Y			+++
	5006-3	*Lactobacillus sp (gasseri,acidophilus)*	Sheep Blood	MRS	35	Y			+++
	5006-4	*Lactobacillus sp (gasseri,acidophilus)*	Sheep Blood	MRS	35	Y			+++
	5006-5	*Lactobacillus sp (gasseri,acidophilus)*	Sheep Blood	MRS and TSA+ (low biomass)	35	**N**	with additives		++
5007	5007-1	*n/a; diphtheroids*	Sheep Blood	BHI+ (low biomass)	35	Y	+	+++	-
	5007-2	*n/a; diphtheroids*	Sheep Blood	BHI+	35	Y	+	+++	-
5008	5008-1	*Klebsiella pneumoniae*	Sheep Blood	MRS and TSA+	35	Y	with additives		+++
	5008-2	*Streptococcus anginosus*	Sheep Blood	MRS and TSA+	35	Y	with additives		+++

	5008-3	*Lactobacillus sp (gasseri,acidophilus)*	Sheep Blood	MRS and TSA+	35	Y	with additives		+++
5010	5010-1	*Klebsiella pneumoniae*	Sheep Blood	MRS	35	Y			+++
	5010-2	*Lactobacillus delbrueckii*	Sheep Blood	MRS	35	Y			+++
5011	5011-1	*Enterococcus faecalis*	CN anaerobic	MRS	35	Y	++	+	+++
	5011-2	*Lactobacillus sp (acidophilus,gasseri)*	Sheep Blood	MRS	35	Y	++	+	++++
	5011-3	*Lactobacillus sp (casei, rhamnosus,paracasei)*	Sheep Blood	MRS	35	Y	++	+	+++
5012	5013-1	*Staphylococcus epidermidis*	Sheep Blood	BH	35	Y	+++	+++	+++
	5013-2	*Staphylococcus ludgenensis*	Sheep Blood	MRS	35	Y	+++	-	+++
	5014-1	*Lactobacillus jensenii*	Sheep Blood	MRS	35	Y	-	-	+++
5014	5015-1	*Streptococcus anginosus*	Sheep Blood	MRS	35	**N**	-	-	++
5016	5016-1	*Aerococcus urinae*	Sheep Blood	MRS	35	Y	-	-	+++
	5016-2	*Lactobacillus crispatus*	Sheep Blood	MRS (low biomass)	35	**N**			+
	5016-3	*Enterococcus faecalis*	Sheep Blood	MRS	35	Y			+++
5017	5017-1	*Escherichia coli*	Sheep Blood	MRS	35	Y	+++	+++	+++
	5017-2	*Escherichia coli*	Sheep Blood	MRS	35	Y	+++	+++	+++
5013	5018-1	*Staphylococcus epidermidis*	Sheep Blood	MRS	35	Y	+++	+++	+++
5031	5001r-1	*Lactobacillus sp (casei, rhamnosus,paracasei)*	Sheep Blood	MRS	35	Y			+++
5008r	5008r-1	*Lactobacillus spp strain 1*	Sheep Blood	MRS	35	Y	-	+	+++

	5008r-2	*Lactobacillus spp strain 2*	Sheep Blood	MRS	35	Y	-	-	+++
	5008r-3	*Candida albicans*	Sheep Blood	MRS	35	Y	+	++	+++
5023	5023-1	*Streptococcus anginosus*	Sheep Blood	MRS	35	**N**	++	+++	+++
5024	5024-1	*Escherichia coli*	Sheep Blood	MRS	35	Y	+++	+++	+++
	5024-2	*Enterococcus faecalis*	Sheep Blood	MRS	35	Y	+	++	+++
	5024-3	*Lactobacillus sp (casei, rhamnosus,paracasei)*	Sheep Blood	MRS	35	Y			+++
	5024-4	*Candida albicans*	Sheep Blood	MRS	35	Y			+++
	5024-5	*n/a; anaerobic 1*	CN anaerobic	MRS	35	**N**	-	-	++
	5024-6	*n/a; anaerobic 2 - 6A - gray*	CN anaerobic	MRS	35	**N**	-	-	+++
	5024-7	*Lactobacillus jensenii*	Sheep Blood	MRS	35	Y	-	-	+++
	5024-8	*n/a; anaerobic 2 - 6B - white*	CN anaerobic	MRS	35	**N**	+	++	++
5025	5025-1	*Enterococcus faecalis*	Sheep Blood	MRS	35	Y	++	++	+++
5026	5026-1	*Corynobacerium coyleae*	Sheep Blood	BHI (low biomass)	35	**N**	-	+	-
	5026-2	*Staphylococcus epidermidis*	Sheep Blood	MRS	35	Y	++	++	+++
	5026-3	*Lactobacillus crispatus*	Sheep Blood	MRS (low biomass)	35	**N**	-	-	1/2+
	5026-4	*Staphylococcus hominis*	Sheep Blood	MRS	35	Y	+	+	+++
5028	5028-1	*Lactobacillus delbrueckii*	Sheep Blood	MRS	35	Y	-	-	+++
	5028-2	*Klebsiella pneumoniae*	Sheep Blood	MRS	35	Y	++	+++	+++

5036	5005r-1	*Proteus mirabilis*	Sheep Blood	BHI	35	Y	++	++	++
5010r	5010r-1	*Candida glabrata*	yeast medium	MRS	35	Y	++	++	+++
	5010r-2	*Escherichia coli*	Sheep Blood	BHI	35	Y	++	++	++
5029	5029-1	*Lactobacillus crispatus*	Sheep Blood	MRS (2 days)	35	**N**	-	-	+++
5006r	5006r-1	*Lactobacillus sp (acidophilus, gasseri)*	Sheep Blood	MRS	35	Y	-	-	++++
	5006r-2	*Streptococcus anginosus*	Sheep Blood	MRS	35	Y	-	++	+++
5000r	5000r-1	*Raoultella ornithinolytica*	Sheep Blood	MRS	35	Y	+	+	+++
	5003r-1	*Lactobacillus iners*	Sheep Blood	TSA+blood (2-3 days)	35	**N**	-	-	-
5009r	5009r-1	*Lactobacillus crispatus*	Sheep Blood	NYCIII+blood	35	Y	-	-	-
	5009r-1	*Lactobacillus crispatus*	Sheep Blood	MRS+blood,Hemin/K	35	**N**			
5049	5017r-1	*Streptococcus bovis group*	Sheep Blood	MRS	35	Y	+	+++	+++
5002r	5002r-1	*Lactobacillus delbrueckii*	Sheep Blood	MRS	35	Y			+++
	5002r-2	n/a; diphtheroids	Sheep Blood	MRS	35	Y			+++
	5002r-3	*Aerococcus urinae*	Sheep Blood	BHI+blood,Hemin/K	35	Y	-	-	1/2+
	5002r-3	*Aerococcus urinae*	Sheep Blood	TSA	35	**N**	+	-	-
5027	5027-1	*Escherichia coli*	Sheep Blood	MRS	35	Y	+++	+++	+++
5030	5030-1	*Leuconostoc sp*	Sheep Blood	BHI, TSA	35	**N**	++	++	-
	5030-2	*Streptococcus anginosus*	Sheep Blood	MRS	35	Y	++	++	+++

5007r	5007r-1	*Streptococcus sp (salivarius ssp thermophilus, vestibulare, salivarius ssp salivarius)*	Sheep Blood	MRS	35	Y	++	++	+++
5037	5037-1	*Lactobacillus sp (gasseri, acidophilus)*	Sheep Blood	MRS	35	Y	-	-	+++
5038	5038-1	*Klebsiella oxytoca*	Sheep Blood	MRS	35	Y	+++	+++	+++
	5038-2	*Lactobacillus sp (rhamnosus, casei, paracasei)*	Sheep Blood	MRS	35	Y	+++	+++	+++
5039	5039-1	*Staphilococcus epidermidis*	Sheep Blood	MRS	35	Y	+++	+++	+++
	5039-2	*Streptococcus sanguinis alpha*	Sheep Blood	MRS	35	Y	+++	++	+++

Notes:

As described, this repository was collected by propagating EQUC-isolated bacterial samples from Vaughan et al, 2021 study.

n/a = data not available; Aerobic culturing was done without agitation while Anaerobic culturing was done using anaerobic gas-pak with aerobic inoculations.

Symbols in the last three columns indicate the qualitative amount of growth observed, ranging from no growth (-) to the most robust growth (+++).

Table S2. A subset of urinary lactobacilli isolates growing under aerobic* MRS conditions.

Isolate ID	MALDI-TOF identified species	Sanger 16s rRNA gene identity	Patient Cohort
5006-2	*Lactobacillus sp (gasseri,acidophilus)*	*Lactobacillus gasseri*	Post-menopausal on estrogen [no UTI]
5006-3	*Lactobacillus sp (gasseri,acidophilus)*	*Lactobacillus gasseri*	Post-menopausal on estrogen
5006-4	*Lactobacillus sp (gasseri,acidophilus)*	*Lactobacillus gasseri*	Post-menopausal on estrogen
5008-3	*Lactobacillus sp (gasseri, acidophilus)*	*Streptococcus anginosus*	Recurrent UTI on vaginal estrogen only [rUTI]
5010-2	*Lactobacillus delbrueckii*	*Lactobacillus delbrueckii*	Recurrent UTI on vaginal estrogen only
5011-2	*Lactobacillus sp (acidophilus, gasseri)*	*Lacticaseibacillus rhamnosus*	Post-menopausal on estrogen
5011-3	*Lactobacillus sp (casei, rhamnosus,paracasei)*	*Lacticaseibacillus rhamnosus*	Post-menopausal on estrogen
5014-1	*Lactobacillus jensenii*	*Lactobacillus jensenii*	Recurrent UTI on vaginal estrogen only
5024-3	*Lactobacillus sp (casei, rhamnosus, paracasei)*	*Lacticaseibacillus rhamnosus*	Recurrent UTI on vaginal estrogen and antibiotics [rUTI+ab]
5024-7	*Lactobacillus jensenii*	*Lactobacillus jensenii*	Recurrent UTI on vaginal estrogen and antibiotics
5028-1	*Lactobacillus delbrueckii*	*Lactobacillus delbrueckii*	Recurrent UTI on vaginal estrogen only
5037-1	*Lactobacillus sp (gasseri, acidophilus)*	*Lactobacillus johnsonii*	Post-menopausal on estrogen
5038-2	*Lactobacillus (rhamnosus, casei, paracasei)*	*Lacticaseibacillus rhamnosus*	Recurrent UTI on vaginal estrogen and antibiotics
5001r-1	*Lactobacillus sp (casei, rhamnosus,paracasei)*	*Lacticaseibacillus rhamnosus*	Recurrent UTI on vaginal estrogen only
5002r-1	*Lactobacillus delbrueckii*	*Lactobacillus delbrueckii*	Recurrent UTI on vaginal estrogen and antibiotics
5006r-1	*Lactobacillus so (acidophilus, gasseri)*	*Lactobacillus gasseri*	Post-menopausal on estrogen
5008r-1	*Lactobacillus spp strain 1*	*Ligilactobacillus animalis*	Recurrent UTI on vaginal estrogen only
5008r-2	*Lactobacillus spp strain 2*	*Ligilactobacillus animalis*	Recurrent UTI on vaginal estrogen only
5016-2	*Lactobacillus crispatus*	*Lactobacillus crispatus*	Post-menopausal on estrogen
5029-1	*Lactobacillus crispatus*	*Lactobacillus crispatus*	Post-menopausal on estrogen

* aerobic – growing statically in ambient atmospheric condition but without active aeration via shaking or rolling

Table S3. Co-occurrence of commensal urinary lactobacilli with pathobionts in the same urine sample.

ID	Bacterial Isolate	Cohort
5010-2<br>5010-1	*L. delbrueckii*<br>*K. pneumoniae*	Recurrent UTI on vaginal estrogen only
5011-2<br>5011-3<br>5011-1	*L. rhamnosus*<br>*L. rhamnosus*<br>*E. faecalis*	Post-menopausal on estrogen
5016-2<br>5016-1	*L. crispatus*<br>*E. faecalis*	Post-menopausal on estrogen
5024-3<br>5024-7<br>5024-1<br>5024-2	*L. rhamnosus*<br>*L. jensenii*<br>*E. coli*<br>*E. faecalis*	Recurrent UTI on vaginal estrogen only
5028-1<br>5028-2	*L. delbrueckii*<br>*K. pneumoniae*	Recurrent UTI on vaginal estrogen only
5038-2<br>5038-1	*L. rhamnosus*<br>*K. oxytoca*	Recurrent UTI on vaginal estrogen only

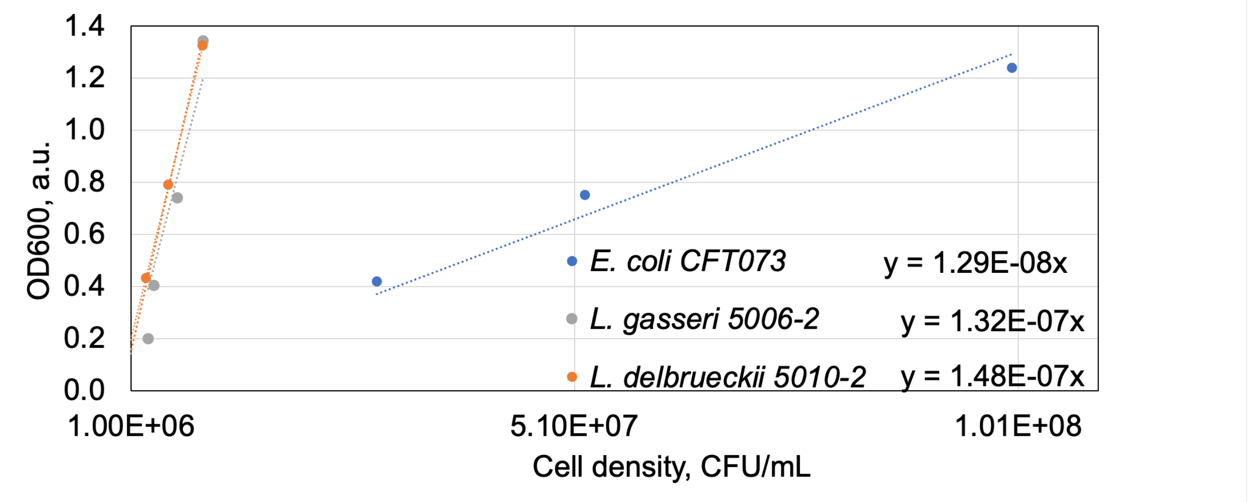

Figure S1. Estimating the relationship of colony forming units (CFU/mL) with turbidity (OD600). *Comparison of the calibration curves show that under similar OD600 turbidity, E. coli CFT073 has higher viable cell density than L. gasseri 5006-2 or L. delbrueckii 5010-2.*

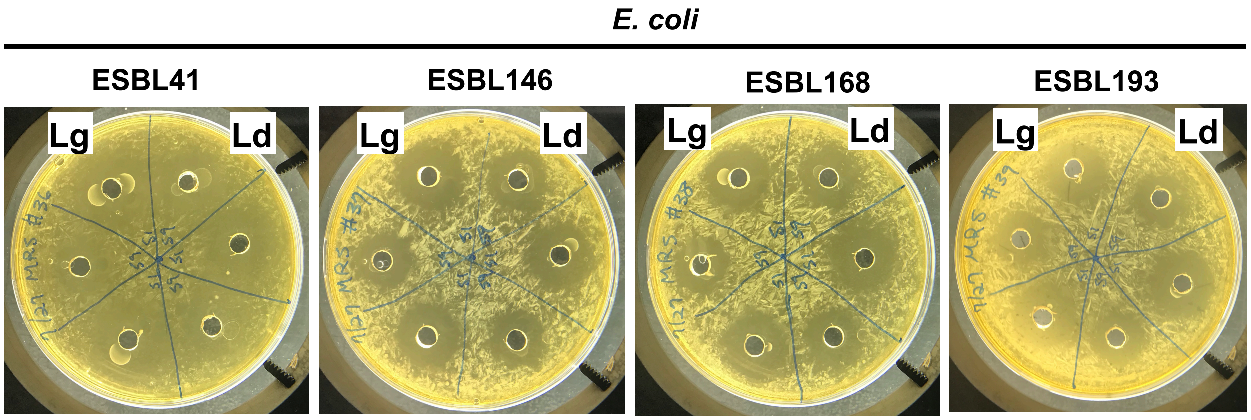

Figure S2. Clinical multidrug resistant E. coli isolates are inhibited by urinary lactobacilli. *Well-diffusion inhibition assays using extended-spectrum beta-lactamase producing E. coli strains ESBL41, ESBL146, ESBL168 and ESBL193*[42] *respectively with two-day old cultures of L. gasseri 5006-2 (Lg) and L. delbrueckii 5010-2 (Ld) loaded into wells.*

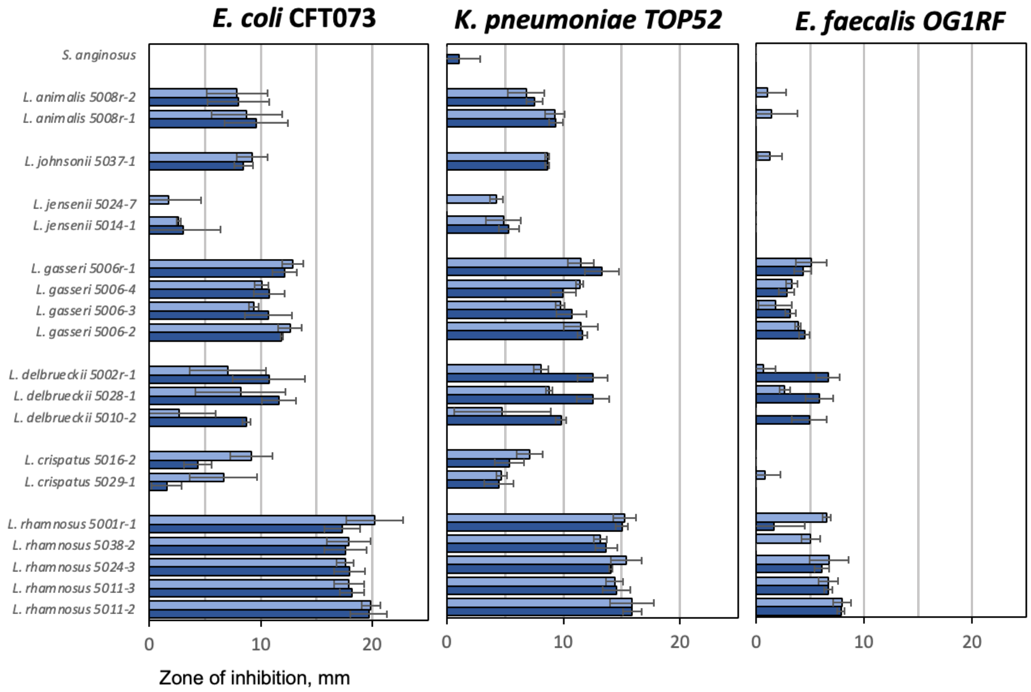

Figure S3. Screening urinary lactobacilli cultures for inhibition of three major uropathogens. *Each lactobacilli MRS culture was tested for its ability to inhibit the growth of E. coli CFT073, K. pneumoniae Top52, and E. faecalis OG1RF in well-diffusion assay at 24 and 48 hrs of growth. Major biomass increase is happening at first 24 hrs for the most cultures.*

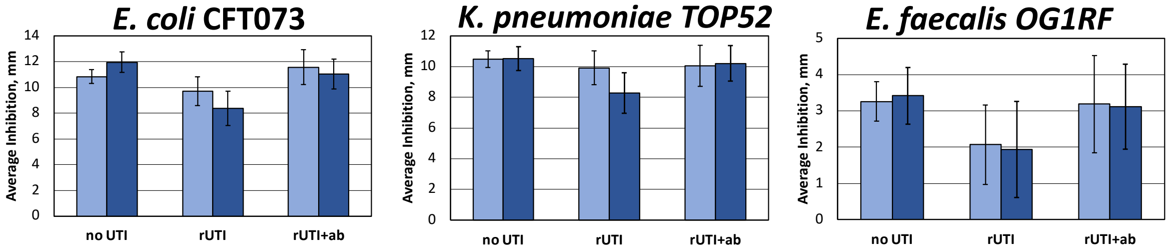

Figure S4. Averaged zones of inhibition of major uropathogens per patient cohorts. *Screen of 19 urinary lactobacilli strains was done for 24 (light blue) and 48 (dark blue) hr MRS cultures and three uropathogens. Zones of inhibitions were averaged amongst three patient cohorts (Table S3). No statistically significant differences (t-test) are observed for such interspecies mean inhibition.*

A

B

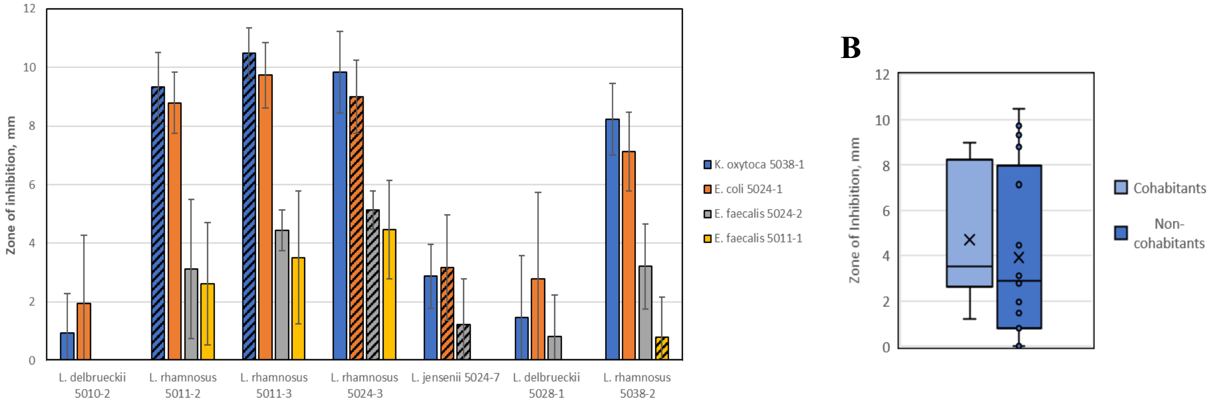

Figure S5. Lactobacilli and pathobiont species found together in the bladder do not inhibit each other more than those found separately.

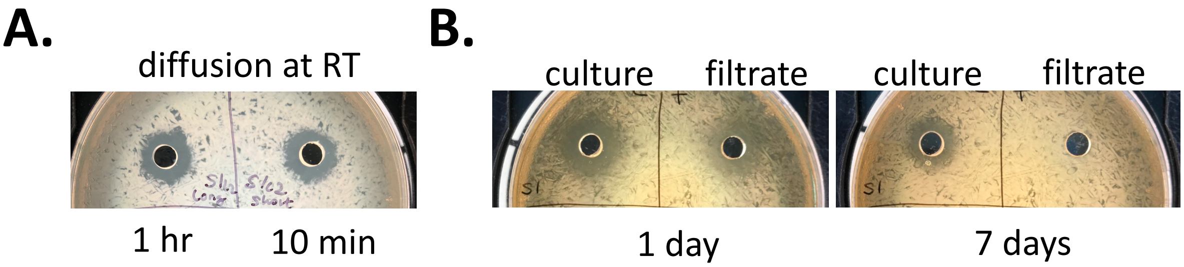

Figure S6. Different behavior of inhibition zones induced by washed cells and cell-free filtrates. ***A.*** *Control experiment showing the well-diffusion outcome is not changing up until 1 hour incubation of the loaded solution at room temperature (RT). This test was done to ensure that longer diffusion at RT while the lawn E. coli bacteria are not growing does not bias the results.* ***B.*** *A plate of CFT073 lawn that interacts with L. gasseri 5006-1 culture or cell-free filtrate with the photographs taken 1 day of incubation at 37°C and ~6 days at ambient temperature.*
